# The persuasiveness puzzle about bootstrapping

**DOI:** 10.1111/rati.12253

**Published:** 2020-01-06

**Authors:** Guido Melchior

**Affiliations:** ^1^ Department of Philosophy University of Graz Heinrichstraße 26 8010 Graz Austria; ^2^ Department of Philosophy Alpen‐Adria‐Universität Klagenfurt Universitätsstraße 65–67 9020 Klagenfurt Austria

**Keywords:** bootstrapping, doubting, Mooreanism, persuasiveness, reasoning

## Abstract

This paper aims at resolving a puzzle about the persuasiveness of bootstrapping. On the one hand, bootstrapping is not a persuasive method of settling questions about the reliability of a source. On the other hand, our beliefs that our sense apparatus is reliable is based on other empirically formed beliefs, that is, they are acquired via a presumably complex bootstrapping process. I will argue that when we doubt the reliability of a source, bootstrapping is not a persuasive method for coming to believe that the source is reliable. However, when being initially unaware of a source and its reliability, as in the case of forming beliefs about our sense apparatus, bootstrapping can be eventually persuasive.

## 1 BOOTSTRAPPING

Vogel (2000, 2008) introduces bootstrapping by presenting the following case. Roxanne looks at her gas gauge repeatedly, drawing the following inductive inference:

**Gas gauge**
  1. Tank is full at t_1_ (Reading the gauge)
  2. Gauge reads ‘F’ at t_1_ (Perception)
  3. Gauge reads ‘F’ at t_1_ & Tank is full at t_1_ (Logical inference)
  4. Gauge reads accurately at t_1_ (Logical inference)
  5. Repeat […]
  6. Gauge is reliable (Induction)

The case of Roxanne is an example of bootstrapping where a subject uses information delivered by a source to conclude that the source is reliable. This is the general pattern of bootstrapping reasoning:

**Bootstrapping**
P_1_: *p*_1_
P_2_: * O* indicates that *p*_1_
P_3_: *p*_1_ ∧ (*O* indicates that *p*_1_)
Repeat for *p*_2_…*p*_n_
Conclusion: *O* is reliable1Bootstrapping is usually understood as a process of inductive reasoning to the conclusion that the target source is reliable. However, one can also formulate a version of deductive bootstrapping, e.g. by adding the premise that *p*_1_–*p*_n_ are all deliverances of *O*. See Melchior (2016). The following reflections equally concern inductive and deductive bootstrapping.

One reason for the philosophical significance of bootstrapping is that Mooreanism is an instance of bootstrapping. I understand Mooreanism as an anti–skeptical strategy of reasoning that one's own sense apparatus is reliable and that one is not in a skeptical scenario by relying on information delivered by one's sense apparatus. Here is a simple version of Moorean bootstrapping2I will discuss more complex forms of Moorean bootstrapping that involve further background knowledge below.:

**Moorean bootstrapping**
  1. *p*_1_ at t_1_ (via perception)
  2. I believe via perception that *p*_1_ at t_1_
  3. I believe via perception that *p*_1_ at t_1_ & *p*_1_ at t_1_ (via deductive inference)
  4. My perceptual belief that *p*_1_ at t_1_ is true (via deductive inference)
  5. Repeat […]
  6. My sense apparatus is reliable (via inductive inference)
  7. The skeptical hypothesis is false (­*sh*) (via deductive inference from (6))

We face conflicting views on the epistemic appropriateness of bootstrapping. On the one hand, bootstrapping is intuitively an epistemically defective reasoning process and, accordingly, it is often assumed that it cannot yield knowledge or justification about the reliability of the target source. Notably, there is wide agreement that bootstrapping is epistemically defective, but there are various rival explanations on the market of why it is defective.3For an overview, see Weisberg (2012). According to one strategy, bootstrapping is flawed because it can only deliver the result that the target source is reliable, regardless of whether it is reliable or not. Weisberg (2012) labels this the no risk, no gain diagnosis. For defenders of this strategy, see Titelbaum (2010), Douven and Kelp (2013). I argue in Melchior (2019) that sensitivity is necessary for checking but not for knowing. Since bootstrapping is an insensitive method it is a flawed method for checking but this insensitivity does not automatically rule out that it is a correct method for acquiring knowledge. On the other hand, bootstrapping is defended as being epistemically flawless, at least under certain circumstances. Bergmann (2004, 2006), for example, distinguishes between unquestioned source situations or contexts and questioned source situations or contexts. In the first case, the person neither is nor should be questioning or doubting the reliability of a source. In the second case, she *is or should be* doing so. Bergmann argues that bootstrapping is benign in the first context but malignant in the second.

One type of explanation of the epistemic defectiveness or of the alleged defectiveness of bootstrapping focuses on bootstrapping's lack of persuasiveness. Some authors, such as Markie (2005) and Lynch (2010, 2012) point out that bootstrapping is not a persuasive way of arguing. Markie (2005) defends bootstrapping by arguing that it *seems* to be a defective reasoning process because it begs the question in argumentative situations, but it actually is a flawless method of knowledge acquisition. Pryor (2004) argues similarly that Moorean arguments are ‘persuasively crippled’, but their justificatory structure is flawless. Cohen (2005) responds to Markie that bootstrapping is not only dialectically inefficient, it is also not a persuasive way of reasoning for oneself.

Reasoning as a cognitive process that a single person undergoes and argumentation understood as an interaction between two interlocutors are structurally similar in that they both proceed from some premises to a conclusion. In this paper, I will focus on persuasiveness (or lack of persuasiveness) of bootstrapping *reasoning*, leaving an investigation of bootstrapping and persuasive argumentation, where similar but slightly different mechanisms are in play, for another paper.

Persuasiveness concerns belief forming mechanisms and is in this respect a psychological phenomenon. We must distinguish psychological questions as those concerning persuasiveness from epistemic questions about whether warrant transmits via bootstrapping and whether bootstrapping can yield justification or knowledge. In this paper, I will first focus on the psychological aspects of the unpersuasiveness of bootstrapping, turning later to consider the epistemic implications for warrant transmission.

We saw that there are conflicting intuitions on the market on whether bootstrapping can yield justification and knowledge. This conflict also carries over to our practice of forming beliefs and leads to a puzzle about belief formation via bootstrapping. On the one hand, if we raise the question whether a source *O* is reliable, we usually do not come to believe that *O* is reliable via bootstrapping reasoning. For example, if Roxanne raises the question whether her gas gauge is reliable, she usually does not settle this question by coming to believe that it is reliable by repeatedly looking at it. Rather she suspends judgment about the reliability of her gas gauge and about the truth of the information that it delivers until she receives further information via an independent source, for example, by using a dip stick. On the other hand, we all believe, at least outside philosophical contexts, that we are normal persons and not brains in vats. However, we do not form these beliefs merely via a priori reasoning and these beliefs are not innate. Rather we form them on the basis of empirical beliefs about objects in the world and about our sense apparatus and based on background information about its reliability, which is also empirically acquired. Hence, we believe via a complex form of Moorean bootstrapping that we are not brains in vats. We then face a puzzle about the persuasiveness of bootstrapping, resulting from our conflicting practices of belief formation. Call this the *persuasiveness puzzle of bootstrapping*. Explaining this puzzle is the primary target of this paper. We can distinguish explaining a philosophical puzzle, which means to explain how conflicting intuitions arise, from solving a philosophical puzzle, which means to take a stance on which intuitions are true and which ones are false. After attempting such an explanation, I will briefly reflect on the implication for existing solutions to the bootstrapping problem.

The argumentation of this paper is based on two hypotheses: Both hypotheses are empirically supported, at least in an indirect way.4To the best of my knowledge, there is no empirical study that directly investigates these two hypotheses. The first hypothesis holds that we come to believe that our sense apparati are reliable via bootstrapping reasoning when we are initially unaware of our sense apparati. This hypothesis is empirically uncontroversial. Our beliefs that our sense apparati are reliable are not innate. Empirical evidence from investigations in the field of metacognition shows that, in their cognitive development, children form beliefs about the external world in the first year. However, they acquire the capacity for meta–representation, which is necessary to form full–fledged beliefs about their sense apparati and their reliability, at the age of around four years.5For a thorough investigation of how and when we acquire concepts of representation in our cognitive development, see Perner (1991). For investigations of metacognition from various angles, see Beran et al. (2012). It has recently been challenged that the exercise of metacognitive capacities requires the possession of meta–representations. See Sodian et al. (2012). Goupil and Kouider (2019), among others, argue against the established view that children acquire metacognitive capacities later in their cognitive development at the age of about four. They argue that new investigations based on non–verbal behaviour including error monitoring and decision confidence show that even preverbal infants have metacognitive capacities. They suggest that this core metacognition is an innate module. However, they admit that this core metacognition must be distinguished from explicit metacognition, which depends on the development of higher–order cognitive functions and culturally situated learning. Importantly, this explicit metacognition, which involves an understanding of the representational mind, is crucial for acquiring beliefs in the reliability of our sense apparati. These beliefs are not innate but acquired via a complex form of bootstrapping reasoning. Hence, they are initially unaware of their sense apparati and form beliefs in the reliability of their sense apparati later in their cognitive development. Moreover, these beliefs are plausibly formed (and sustained) on the basis of experiences and not via any a priori reasoning. Hence, we form in our cognitive development beliefs in the reliability of our sense apparati via a complex form of bootstrapping reasoning when we are initially unaware of our sense apparati. The second hypothesis states that we tend not to believe on the basis of bootstrapping reasoning that a source is reliable when raising the question about the reliability of a source. This hypothesis is underpinned by our empirically observable hesitation, also manifested in philosophical discussion on bootstrapping, to form beliefs about the reliability of sources via obvious bootstrapping reasoning. In this respect, both hypotheses fit well with empirical evidence.

## 2 BELIEVING AND REASONING

Let me start with some remarks on doxastic attitudes. A subject S can have different doxastic attitudes towards a proposition *p*. S can believe that *p*, S can reject that *p*, and S can suspend judgment about *p*.6For the sake of simplicity, I will assume here that S rejects that *p* iff S believes that *p*. However, nothing hinges on this assumption. Moreover, I will assume for simplicity reasons that credences can be mapped onto this tri–partite structure. In each of these three cases, S has a doxastic attitude towards *p*, which implies grasping the concepts that constitute *p*. However, these three alternatives are not exhaustive since a subject can also lack any doxastic attitude towards a proposition. For example, a child does not have any doxastic attitude towards Fermat's last theorem, since she does not grasp the concepts that are involved in this proposition. I will call lack of any doxastic attitudes towards *p* ‘unawareness of *p*.’ Being unaware about *p* can be distinguished from having an implicit doxastic attitude towards *p*. In both cases, the subject does not actually entertain *p* and might never have entertained *p*. For example, a person sitting in London might never have entertained the proposition that there are more than 13 bicycles in Shenzhen. However, when confronted with this proposition, she will affirm it, reject it, or suspend judgment about it. In contrast, a person who is unaware of this proposition might also fail to affirm or reject this proposition or to suspend judgment about it when confronted with it.

Let me introduce some terminology: Doubting that *p* is, according to our natural language understanding, a non–positive doxastic attitude towards *p*. I will define doubting as follows:

**Definition: doubting**
S *doubts that p* iff S rejects that *p* or suspends judgment about whether *p* is true.

Doubting that *p* requires a doxastic attitude towards *p*, either a neutral one in case of judgement suspension or a negative one in case of rejection. Lack of awareness, which does not involve any doxastic attitude towards *p*, is by definition not an instance of doubting. The provided definition of doubting is presumably not entirely in line with our natural language understanding, which regards doubting rather as suspending judgment about a proposition than rejecting it. However, for the sake of terminological simplicity I will use ‘doubting’ as defined here.

Reasoning is a potential belief forming method among others, such as perception or testimony. However, reasoning is not always the original cause of a belief. A person might already believe that *p* and then confirm that *p* via reasoning. I assume that reasoning starts with one or more premises and reaches a conclusion.7For the sake of simplicity, I assume here that any reasoning process leads to exactly one conclusion. Let me start with a general assumption about reasoning as a belief forming process, which is captured by the following principle:

**PCB**
S usually believes that *c* because of a reasoning process *P* consisting of premises *p*_1_…*p*_n_ and conclusion *c* only if S believes premises *p*_1_…*p*_n_.8One might object that there are exceptions to PCB. For example, I might come to believe the conclusion of a reductio argument by hypothetically assuming the premises. However, I would argue that reductio arguments confirm to PCB once their premises are spelled out in detail.

PCB is an empirical hypothesis that only claims that we usually follow the rule of not believing the conclusion of an argument because of the argument if we do not believe the premises. For sure, we do not always follow this rule. In fact, we are fallible reasoners and often violate various rules of rationality in various ways, including presumably the rule corresponding to PCB.9Plausibly, we not only usually but also reasonably behave this way. However, the focus of this paper is the psychological mechanism of belief formation. Hence, I will not commit myself to reflecting on this additional assumption.


## 3 BOOTSTRAPPING REASONING

Let me now address particular beliefs about the reliability of sources. We can take three doxastic attitudes towards a proposition that a particular source *O* is reliable – we can believe it, reject it, or suspend judgment about it. There is a connection between believing in the reliability of *O* and believing information delivered by a source *O*. I assume this connection is such that when we reject that *O* is reliable or suspend judgment about its reliability, then we usually, though not necessarily, suspend judgment about the truth of information which we think is delivered by *O*. This connection is captured by the following principle:

**DR**
If S doubts that *O* is reliable and believes that information *i* is delivered by *O*, then S *usually* suspends judgment about the truth of *i*.

Let me make some clarificatory remarks. (1): For suspending judgment about the truth of information *i*, it is crucial that we *believe* that *i* is delivered by a source *O* whose reliability we doubt. We also suspend judgment about the truth of *i* if we falsely believe that *i* is delivered by another source *O*' whose reliability is also in doubt. Moreover, *i* might be delivered by *O*, but if we do not believe that it is, we do not automatically suspend judgment about the truth of *i*. (2): Suspending judgment about the truth of *i* depends on *doubting* that *O* is reliable, it does not depend on whether *O* is actually unreliable. Again, we might be mistaken in our doubt that *O* is reliable yet nevertheless suspend judgment about the truth of *i*. (3): If we believe that *i* is delivered by a source whose reliability we doubt, then we usually suspend judgment about the truth of *i instead of believing* in the falsity of *i*. We usually only form this belief if we have a stronger belief about *O*, for example, that *O* always, not only often, delivers false information. (4): The principle DR is restricted to doubting the reliability of *O*, that is, rejecting that *O* is reliable or suspending judgment about its reliability. It does not include *being unaware* that *i* is delivered by *O*. We will see, that DR cannot be extended to unawareness. If S is unaware of *O* being reliable, then S might still believe that *i* via *O*. (5): DR is only a claim about usual belief forming mechanisms. It does not say that this behaviour is line with norms of rationality. One might think that subjects not only usually but also *reasonably* suspend judgment about the truth of information of which they think that it delivered by a source whose reliability they doubt.10See Pryor (2004) who distinguishes questions of justification transmission from questions of what one rationally believes given that she has certain beliefs. According to this distinction, S can be justified in believing about some source that it is reliable via bootstrapping reasoning, although it might be rational for her to suspend judgment about the truth of information delivered by this source, given that S doubts its reliability. As we will see in the last section, things here become tricky if we take the epistemic consequences of the bootstrapping problem into account.

So far, we have introduced two principles, PCB and DR. We can now see how these principles jointly determine that we usually do not believe via bootstrapping reasoning if we doubt the reliability of the target source.

**The argument against the persuasiveness of bootstrapping**
  1. S usually believes that *c* because of a reasoning process *P* consisting of premises *p*_1_…*p*_n_ and conclusion *c*, only if S believes premises *p*_1_…*p*_n_. (PCB)
  2. If S doubts that *O* is reliable and believes that information *i* is delivered by *O*, then S usually suspends judgment about the truth of *i*. (DR)
  3. In case of bootstrapping reasoning, information delivered by *O* constitutes the premises of an argument that *O* is reliable. (Definition of bootstrapping)
  4. If S doubts that *O* is reliable and recognizes a potential reasoning process as an instance of bootstrapping then S does not believe the conclusion that *O* is reliable because of this bootstrapping process. (From 1, 2, and 3)

DR makes a claim about judgment suspension given that we doubt the reliability of the target source. We have seen that the three doxastic attitudes of belief, rejection, and judgment suspension do not exhaustively characterize our possible stances towards a proposition. We can also be unaware about a proposition and thus fail to have any doxastic attitude towards *p* at all. Does DR also hold for unawareness about *O*'s reliability? Arguably it does not. Suppose S is unaware of *O* being a reliable source, because S is unaware that *O* is exists. Take the case of a child who does not have any concept of a sense apparatus or of reliability. Nevertheless, the child forms beliefs by using her sense apparatus. Or take the case of a deceived person who believes to see things that are, unbeknownst to her, projections from a hidden projector of whose existence she is unaware. In both cases, the person is unaware of a source and consequently unaware of its reliability and still believes information delivered by this source. Hence, the following extension of DR does not hold:

The false claim: UAR*
If S is unaware that *O* is a reliable source, then usually S does not believe information delivered by *O*.

Accordingly, NBR* is also false:

NBR*
If S is does not believe that *O* is a reliable source, then usually S does not believe information delivered by *O*.

This outcome is not surprising. DR states that if S doubts that *O* is reliable and believes that information *i* is delivered by *O*, then S *usually* suspends judgment about the truth of *i*. If S is unaware about *O*, then S is not in a position to believe that *i* is delivered by *O* and therefore a requirement for suspending judgment about *i* is not fulfilled.

In cases of unawareness, subjects can come to believe information via a source without believing beforehand that the source is reliable. For our investigation of bootstrapping, it is crucial to settle the following question: Is it also possible that in some of these cases subjects later come to believe *that O is reliable* based on information delivered by *O*? In many cases, this is presumably not the case. For example, if a child believes what she sees on TV without having a concept of a TV, the child plausibly does not come to believe that the TV is reliable via bootstrapping. More plausibly, she will acquire such beliefs via a different path. However, there is at least one case where we acquire a belief about the reliability of a source in a kind of bootstrapping way, namely when it comes to believing in the reliability of our own sense apparati. As children we believe information about the external world delivered by our sense apparati without even having an implicit belief that our sense apparati are reliable. Later in our cognitive and educational development, we form beliefs about our sense apparati and about their reliability.11Holding beliefs about sense apparati and their reliability requires possession of the concept of representations and misrepresentations that are delivered by sense apparati. Perner (1991) shows in thorough empirical investigations that children develop the capacity to reflect about representations at the age of around four years, whereas they form beliefs about external objects based on experiences in their first year. However, we do not form these beliefs a priori. Rather they are at least partly based on information delivered by our sense apparati. Hence, there is at least one case (and there are perhaps other cases as well) where we form beliefs based on information delivered by *O* without having a belief about *O*'s reliability and later form beliefs via a form of (complex) Moorean bootstrapping that *O* is reliable. This complex form of Moorean bootstrapping might involve knowledge about my body, that I did not take any drugs in the past few days, as well as background knowledge about the human sense apparatus, all empirical knowledge acquired via our sense apparati. Notably, this process of Moorean bootstrapping is a process of belief formation but not a process of reasoning that aims at overcoming doubt, namely whether the skeptical hypothesis is true or false during a process of inquiry.

In the introductory section, we formulated a puzzle about bootstrapping. On the one hand, bootstrapping is not a persuasive method for settling a question whether a source is reliable. On the other hand, our belief in the reliability of our own sense apparati is based on a kind of Moorean bootstrapping. We are now in a position to explain this bootstrapping puzzle about belief acquisition. If S doubts that *O* is reliable, that is, if S rejects that *O* is reliable or suspends judgment about it, and believes that information *i* is delivered by *O*, then S *usually* suspends judgment about the truth of *i*. Therefore, bootstrapping is not a persuasive *reasoning* process for overcoming doubts about the reliability of a source. During a typical process of inquiry into whether a source is reliable, we raise the question whether the source is reliable and use a method for settling this question. Hence, we entertain the target proposition whose truth we want to determine. In this case, we are not unaware of the target proposition. Hence, in contexts where we raise the question whether *O* is reliable und use a method for settling this question, bootstrapping is not a persuasive method for us and, consequently, we will not use bootstrapping for settling questions about the reliability of a source. However, the corresponding principle UAR* is false. If S is initially unaware that *O* is a reliable source, then S can believe information delivered by *O*, and in certain cases S can also come to believe that *O* is reliable via bootstrapping when S becomes aware of *O*. Hence, bootstrapping can be a way of forming beliefs. This is the case when we first form beliefs via our sense apparati and later form beliefs about the reliability of our sense apparati that are based on these empirical beliefs.

I have explained why bootstrapping usually fails to be a persuasive process of overcoming doubts even though it can be a successful belief forming process. At this point, one might wonder whether this psychological behaviour is also rational in some relevant way. This paper does not directly address this question, but let me briefly reflect on an account proposed by Pryor (2004) who draws a distinction between justification and what one is rationally committed to believe by beliefs that one already has. He defines rationality as what one ought to believe given the beliefs that one already has independently of whether the beliefs that one has are justified. He argues that the mere belief that a source is not reliable, regardless of whether this belief is justified or not, can rationally obstruct one from believing that information delivered by this source is accurate. Pryor seems inclined to ascribe this obstructing force also to judgement suspension about the reliability of a source. Following Pryor's account, although the justificatory structure of bootstrapping reasoning might be flawless, it is irrational to believe premises of bootstrapping if one doubts that the source is reliable.

Pryor's conception of rationality is a hybrid of subjective and objective features. On the one hand, rationality depends on what one believes regardless of whether these beliefs are justified. On the other hand, there are the same objective rules for all subjects of what they ought to believe given that they hold certain beliefs. In order to explain the puzzle about the persuasiveness of bootstrapping, I focus on what we *usually* believe, which depends on whether we doubt the reliability of a source or not. However, many readers might be inclined to think that this doxastic behaviour also corresponds with an epistemic rule, that is, that we not only usually but also *reasonably* form beliefs along these psychological principles. Those readers might find a satisfactory explanation for their intuitions in Pryor's concept of rationality.

## 4 BOOTSTRAPPING REASONING, KNOWLEDGE AND WARRANT TRANSMISSION

So far, we have mainly focused on the psychological aspects of belief formation. Bootstrapping is not a way of overcoming doubts via reasoning about the reliability of a source, but we might form beliefs that a source is reliable via bootstrapping if we are originally unaware of the source used. In this final section, I will briefly reflect on the implications of these conclusions for questions concerning knowledge and transmission of justification and warrant. In particular, I will sketch the implications for different takes on justification and knowledge, including conservatism and liberalism about justification and contextualism about knowledge. In this paper, I will only investigate some consequences that the results about persuasiveness have for these accounts. However, I will not defend any of these accounts here. For the purposes of this paper, I will remain neutral about central epistemic questions concerning bootstrapping, in particular whether it can yield knowledge and whether justification and warrant can transmit via bootstrapping. Thus, this last section contains some brief remarks rather than a full–fledged theory about knowledge and/or justification via bootstrapping.

Let me first briefly reflect on the relations between bootstrapping reasoning and knowledge acquisition. Can bootstrapping reasoning yield knowledge? Knowledge requires belief. Since S usually does not come to believe via bootstrapping that *O* is reliable if S doubts that *O* is reliable, bootstrapping, for this reason, usually does not yield knowledge in cases of initial doubt that *O* is reliable. In contrast, if S is initially unaware of *O*, then S might believe information delivered by *O* and in some cases eventually come to believe that *O* is reliable via bootstrapping. In this case, bootstrapping is not ruled out as a process of knowledge acquisition for not leading to belief acquisition. Hence, in some contexts bootstrapping reasoning fails to yield knowledge because it fails to yield beliefs, whereas in some other contexts it can yield beliefs, and at least in this respect it is not excluded from yielding knowledge. Let me emphasize at this point that I do not claim here that bootstrapping can yield knowledge. It is a subtle and complex endeavour to settle this question, one that is beyond the scope of this paper. I only claim that, in some contexts, it is not the case that bootstrapping fails to yield knowledge for the particular reason that it fails to yield beliefs. However, I do not claim that there are no other epistemic reasons for why bootstrapping cannot yield knowledge in these contexts, for example because of failure of warrant transmission. In this paper, I remain neutral about these more complex epistemic issues.

Next, let me briefly discuss the implication of the resolution to the persuasiveness puzzle for different theories on whether warrant (or propositional justification) can transmit via bootstrapping reasoning, without taking a stance on which of these theories is right and which one is wrong. Suppose that justification transmits via bootstrapping reasoning. In this case, where S is originally unaware about *O* and then forms a belief that *O* is reliable via bootstrapping, S can have propositional *and doxastic* justification to believe that *O* is reliable. S usually lacks doxastic justification in cases where S doubts that *O* is reliable since S does not believe via bootstrapping, but S has still *propositional* justification that *O* is reliable. Adopting the terminology introduced by Pryor (2004), we can say that liberals about bootstrapping accept that justification transmits via bootstrapping whereas conservatives reject this claim.12This terminology is not entirely in line with Pryor's since he does not define liberalism and conservatism directly in terms of accepting or rejecting bootstrapping reasoning. Liberals, according to his terminology, claim that in absence of any defeaters we can be justified in believing that *p* via source *O* even if we do not have any prior justification about the reliability of *O,* whereas conservatives reject this claim. Liberalism, as defined by Pryor, does not imply that justification transmits via bootstrapping reasoning, see Silins (2008). However, Pryor accepts both views. For a conservative view, see Wright (2002, 2004). According to liberalism, our belief forming process can be in line with the transmission of justification in cases of original unawareness of *O*, whereas we are too cautious in our belief forming processes when doubting that *O* is reliable. According to conservatism, we are epistemically too careless when we are originally unaware of *O* and form beliefs via bootstrapping and act epistemically properly when doubting whether *O* is reliable and not forming beliefs via bootstrapping.13For an overview of potential solutions to the bootstrapping puzzle, see Melchior (2016).


One need not be a liberal or conservative about all sources. Hence, one potential take on the question whether bootstrapping can yield knowledge and/or justification is to treat different sources differently. Pryor (2004, p. 355) and Sosa (2010, p. 138) defend the view that we can have justification or knowledge via our sense apparati without having prior justification or knowledge about their reliability, but they reject this liberal position for other sources such as measurement devices. The paradigmatic beliefs formed via bootstrapping are beliefs about the reliability of our sense apparati. In contrast, we usually do not form beliefs about the reliability of measurement devices via bootstrapping. Hence, the suggestion made by Pryor and Sosa delivers a result for knowledge and justification via bootstrapping that is in line with the fact that we form beliefs via bootstrapping about the reliability of our sense apparati but not about the reliability of other sources. I sympathize with this solution, but one might find the claim of treating different sources differently without providing further arguments ad hoc.

Finally, let me briefly reflect on a potential contextualist take on bootstrapping, which fits well with the provided explanation of the persuasiveness puzzle about bootstrapping. Contextualism about bootstrapping suggests that in certain (ordinary) contexts, we can know via bootstrapping, but in some more demanding contexts, we raise the standards for knowledge up to level such that we cannot know via bootstrapping anymore. Such a contextualist approach fits neatly with our actual belief forming processes if it assumes that if we are originally unaware of *O*, we are in a context that allows for knowledge via bootstrapping, but when doubting whether *O* is reliable, we raise the standards for knowledge up to a level such that bootstrapping cannot yield knowledge anymore.14I discuss but do not endorse such contextualist solutions in Melchior (2019). I regard it as an open question whether contextualism or invariantism provides the correct analysis of ‘know’. Therefore, I remain neutral about whether invariantism or contextualism offers the correct solution of the bootstrapping problem and whether the sketched version of contextualism provides a viable take on knowledge via bootstrapping.

To sum up: Without endorsing a particular solution to the bootstrapping problem, we have seen that the provided analysis of the persuasiveness puzzle of bootstrapping is compatible with different takes on whether one can know via bootstrapping or on whether justification (or warrant) transmits via bootstrapping, contextualist or invariantist.

## 5 CONCLUSION

Concerning bootstrapping, we face a puzzle about persuasiveness. On the one hand, we usually do not overcome doubts about the reliability of a source via bootstrapping. On the other hand, we believe in the reliability of our sense apparatus via bootstrapping, albeit in a complex way. This puzzle can be resolved by distinguishing two reasons for not believing that a source is reliable, namely doubting that a source is reliable and being unaware of a source. Bootstrapping is not a way of overcoming doubts about the reliability of a source, but when we are initially unaware of a source, as we are as children about our own sense apparati, we can eventually come to believe that a source is reliable via bootstrapping. This explanation of the persuasiveness puzzle of bootstrapping is compatible with various takes on whether justification or knowledge can transmit via bootstrapping.
